# Electrochemical Study of Clean and Pre-Corroded Reinforcements Embedded in Mortar Samples with Variable Amounts of Chloride Ions

**DOI:** 10.3390/ma14226883

**Published:** 2021-11-15

**Authors:** María de las Nieves González, María Isabel Prieto, Alfonso Cobo, Fernando Israel Olmedo

**Affiliations:** 1 Escuela Técnica Superior de Edificación, Universidad Politécnica de Madrid, Avda. Ramiro de Maeztu, 7, 28040 Madrid, Spain; mariadelasnieves.gonzalez@upm.es (M.d.l.N.G.); alfonso.cobo@upm.es (A.C.); 2 Valladares Ingeniería S.L, C/Julián Camarillo, 42, 28037 Madrid, Spain; fiolmedoz@gmail.com

**Keywords:** repassivation, corrosion, reinforcement, electrochemical techniques

## Abstract

The present study investigates the possibility of re-surfacing previously corroded reinforcements and the suitability of the two electrochemical techniques that are widely used to determine the state of corrosion of steel (the corrosion potential E_corr_ and the corrosion rate i_corr_). In order to test this, 32 pre-corroded B500SD reinforcing steel bars have been used for one year, where half of the bars have been cleaned to eliminate corrosion products. The other half have been maintained with the generated corrosion products. Subsequently, the bars have been embedded in cement mortar samples with variable amounts of chloride ion, and E_corr_ and i_corr_ have been measured for 250 days. The results showed that it is not possible to rework the reinforcement without removing corrosion products and that it is not possible to predict the passive or active state of steel by measuring E_corr_ only.

## 1. Introduction

Reinforcement corrosion is accepted as the main cause of the reduction in the service life of reinforced concrete structures (RCSs) [1,2,3]. The enormous economic impact of this problem, due to the direct and indirect costs involved, has led to a vast development of new technologies and materials with the purpose of increasing the durability of RCSs [4,5,6,7]. In the USA, direct costs due to corrosion of RCSs infrastructure are estimated at 0.25% of GNP, which corresponds to USD 16.6 billion per year [8].

The steel reinforcements embedded in the RCS are in a passive state that are protected against corrosion. This protection is due to the existence of a passive layer formed at the steel/concrete interface, which is self-healing and very thin around 10 nm [9]. Its formation and stability are guaranteed by the high alkalinity of the concrete, usually between the range of pH 13–14, and by the existence of an appropriate electrochemical potential [10].

The loss of the passivity of the RCS reinforcement is due, in most cases, to the following factors: the presence of despassivating ions, essentially chlorides, in sufficient quantity to locally break up the passivating layers [11]; or the decrease in the pH of the concrete, due to the effect of the CO_2_ present in the atmosphere [12,13,14]. In addition to the triggering factor that induces corrosion, the environment in which the structure is located determines the variables that most significantly influence its behavior. In the case of marine environments, the chloride diffusion coefficient, the concentration of chlorides on the surface and the thickness of the rebar coating are the most determining parameters to be able to evaluate its behavior, while in the case of underground tunnels, although corrosion is induced by chlorides, the formation of NaCl crystals is a parameter to take into account [15,16].

**Figure 1:**
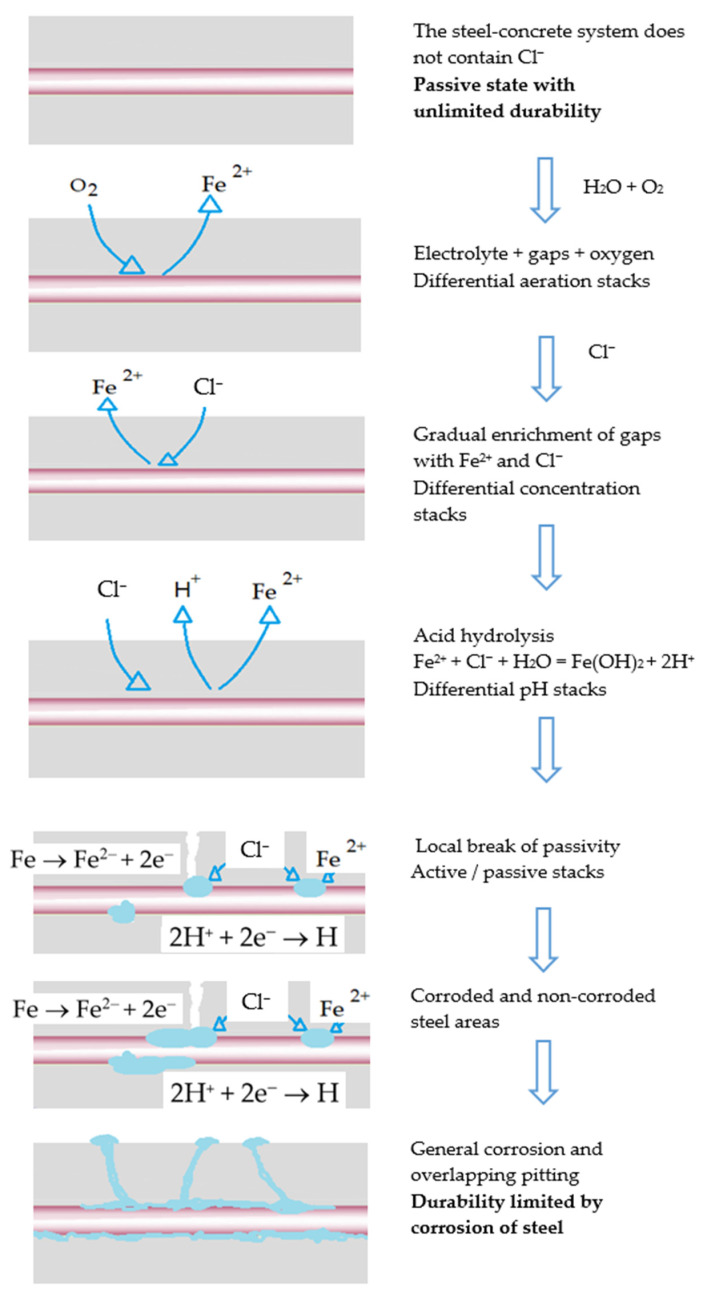
Chloride corrosion mechanism in reinforced concrete structures.

For decades, numerous investigations have been carried out to explain the role of the factors that trigger corrosion of rebars, especially chloride ions [17,18], whose mechanism can be seen in the Figure 1.

The critical threshold of chloride ions (C_crit_) needed to despassivate the steel is one of the critical parameters in the prediction and evaluation of corrosion [17]. The knowledge of C_crit_ is fundamental when establishing the requirements to achieve structures with sufficient durability and to evaluate the service life or residual life of existing structures [18]. The onset of corrosion due to the effect of chlorides in the concrete reinforcements has been related to an increase in the ratio [Cl^−^]/[OH^−^] above a certain value, which was initially set by Haussman at 0.6 [19,20,21,22,23,24]. However, it was later found that it can be affected by other parameters such as the amount of air retained in the holes of the steel-concrete interface or the presence of defects [23,25,26,27,28,29,30]. Numerous studies depict that there is a significant dispersion in C_crit_ values. In most cases, the limits are set in relation to the weight of the cement: For example, for concrete with a water/cement ratio of 0.5 mainly located in marine environments in the intertidal zone in Europe with a limit value of 0.5% suggested [31]. In Spain, the mandatory regulations establish a durability strategy based on the service life of the structure, but always with a limit of Cl^−^ of 0.4% [32]. In the United States, the thresholds are set according to the exposure class of the structure, ranging from 0.15% to 0.30%, and may reach 1% for structures located in dry environments [33]. At present, there are numerous investigations that model the corrosion behavior of the reinforcements embedded in the reinforced concrete and the influence of the concrete quality, the concentration of chlorides and the coating and the cracking, on the rate of corrosion, under different environmental conditions [34,35,36,37]. In addition, these investigations, validated on the corresponding experimental works, have allowed to simulate the influence that the size of the pits have on the geometry of the fissures and how the accumulation of oxide in the pits influence the adhesion between concrete and steel [38].

The realization of potential maps according to ASTM C876-09 [39] is the electrochemical technique most commonly used to diagnose the risk of corrosion in RCSs [2,40]. However, the results of the evaluation of RCSs with corroded reinforcements may indicate different degrees of corrosion or probability of corrosion depending on the technique used for corrosion assessment [41,42]. It is generally accepted that the measurements of corrosion potential (E_corr_) should be completed with other procedures [40,43]. The measurement of corrosion rate is a quantitative technique that is known for decades [44]. However there have been found large variations in the values of corrosion rate for narrow ranges of values of corrosion potential [45].

Taking into account the previous premises, the aims of the work are to verify the possibility of re-surfacing previously corroded reinforcements and to verify the suitability of two electrochemical techniques widely used to determine the corrosion status of reinforcement by correlating the E_corr_ and the i_corr_ measurements.

## 2. Experimental Program

In this study, two issues related to corrosion of RCS reinforcements have been investigated: (i) The possibility of re-surfacing previously corroded reinforcements and (ii) the verification of the suitability of two electrochemical techniques widely used to determine the corrosion status of reinforcements. In order to test this, 32 B500SD reinforcing steel bars of 6 mm diameter and 120 mm length have been used. The chemical composition of the steel is shown in Table 1. The analyses have been carried out with an Optical Emission Spectrometer by Arc/Spark model SPECTROMAX.

The rebars have been embedded in cement mortar samples of 80 × 55 × 20 mm^3^ with a cement/sand/water dosage of 1/3/0.5. CEM I 42.5R Portland cement according to RC-16 standard [46], siliceous sand with a maximum size of 0.4 mm and drinking water supplied by Canal de Isabel II in Madrid were used. The physical characteristics and chemical composition of the cement and the sand used can be seen in Table 2.

**Figure 2:**
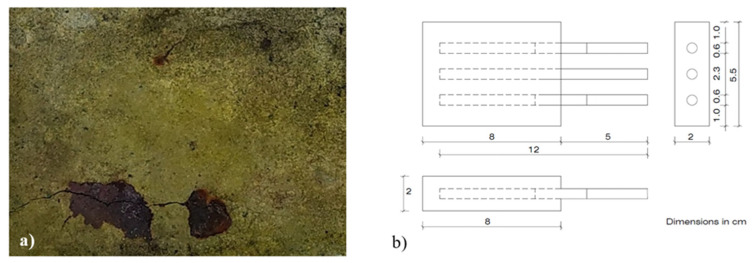
Specimens used in the study of corrosion. (**a**) Symptoms in pre-corroded specimens (first phase); (**b**) Scheme of the specimens with variable amounts of chloride ion (second phase).

The steel rebars were embedded in the specimens, leaving 5 cm outside, in which the risk of differential aeration at the triple atmosphere/mortar/steel interface was eliminated by means of adhesive tape. The specimens were demolded after 24 h and cured in a wet chamber for 28 days at a temperature of 20 ± 2 °C and a humidity above 90%. Subsequently, they were subjected to a constant anodic polarization of 20 µA/cm^2^ during one year. In the photograph (Figure 2a), one of the specimens can be seen after the passage of the electric current. The samples were mixed with an addition of 2% of Cl^−^ in relation to the weight of the cement to ensure that the anodic polarization would lead to corrosion of the reinforcements and would not cause the electrolysis of the water in the pore network.

**Figure 3:**
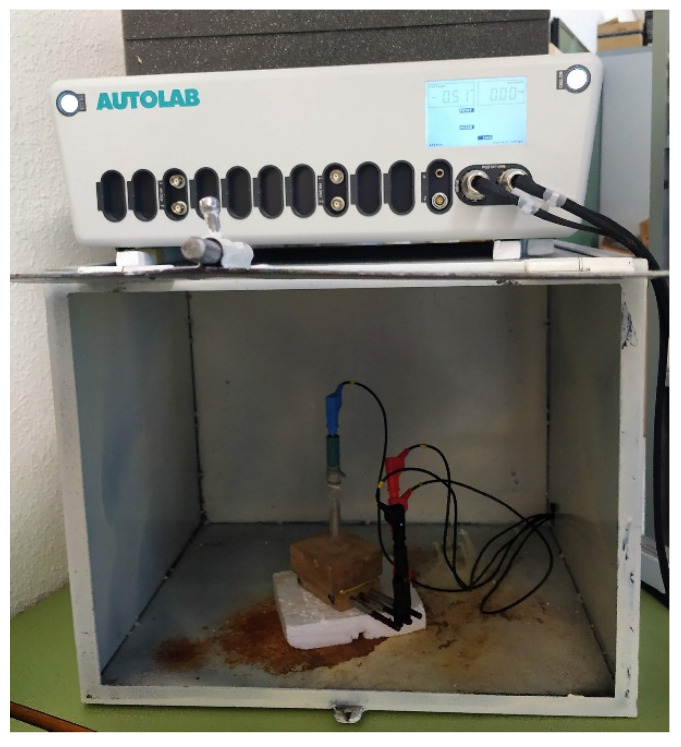
Performing electrochemical measurements.

Once the pre-corrosion of the rebars was complete, the mortar samples were then broken and the rebars removed. Half of the rebars (16 pieces) were chosen at random, and the corrosion products were completely removed by dissolving the iron oxides and hydroxides in 50% HCl inhibited with 4g/l urotropin (hexamethylenetetramine). With the clean rebars (CLN), 8 specimens similar to those of the first phase were performed, but with variable amounts of chloride ion of 0.0%; 0.2%; 0.4%; 0.6%; 0.8%; 1.0%; 1.5%; and 2.0% in relation to the weight of cement and with the incorporation of a third rebar located in the center to facilitate the electrochemical measurements (Figure 2b). The same process has been carried out with the rebars that hold the corrosion products. Then the 16 specimens were kept in a wet chamber for 250 days at a temperature of 20 ± 2 °C and a humidity above 90%. During this time the corrosion potential (E_corr_), and corrosion rate were periodically recorded using an AUTOLAB/PGSTAT302N potentiostat in which the outer bars were used as working electrodes and the central bar as an auxiliary electrode (Figure 3). The reference electrode used was Cu/CuSO_4_. The corrosion rate was measured by the corrosion current density (i_corr_) obtained by measuring the polarization resistance (R_p_) by the Stern and Geary equation [47]:

(1) $$i_{\mathrm{corr}}=\frac{B}{R_{p}}$$

where the value of 26 mV for the constant B was chosen [48]. The value of R_p_ was obtained by applying polarization of 10 mV and measuring the current response after 15 s. The reference values for predicting the corrosion state as a function of E_corr_ and i_corr_ are shown in Table 3 [39,49].

With respect to the thresholds of the E_corr_ value there is a very broad consensus throughout the scientific and technical community, most likely due to the enormous diffusion at the international level of the ASTM C 876-09 standard. Regarding i_corr_ thresholds, it is widely accepted that i_corr_ < 0.1 µA/cm^2^ corresponds to steel in a passive state, and above this value steel corrodes and i_corr_ > 1 µA/cm^2^ is very dangerous [42,44,50,51,52,53].

## 3. Results and Discussion

**Figure 4:**
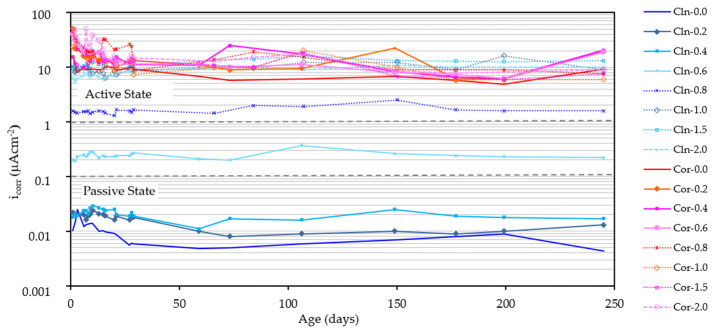
Evolution of i_corr_ over time for all the rebars studied.

**Figure 5:**
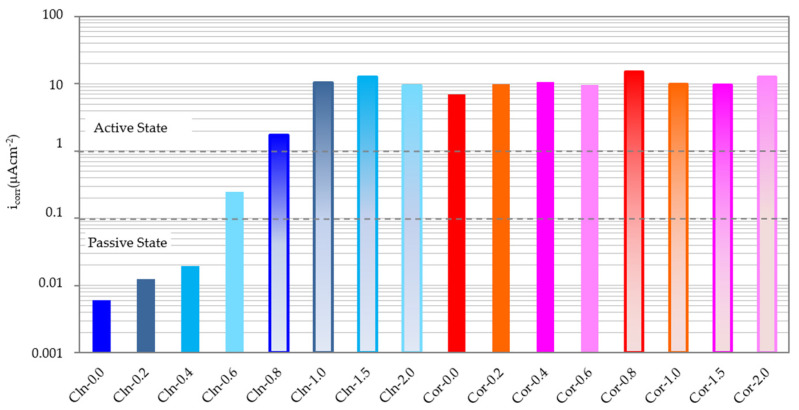
i_corr_ measurement of all rebars after 250 days.

Figure 4 shows the evolution in time of the i_corr_ of all the analyzed rebars. Each of the data have been obtained as the arithmetic mean of the two rebars in the same state and embedded in the same specimen. The abbreviation CLN indicates that the rebar has been cleaned while the abbreviation COR indicates that the rebar keeps the corrosion products. The final numbers indicate the amount of Cl^−^, expressed as a percentage by weight of cement, present in the specimens. It can be seen that, in most cases, the starting point is an i_corr_ that decreases in a very similar way during the first 35 days of exposure. After this period of exposure, the i_corr_ remains at very stable values most probably because of the humidity of the environment remaining constant. Figure 5 shows the values corresponding to the i_corr_ of all the rebars after 250 days.

If the clean rebars (CLN) are analyzed, the analysis depicted in Figure 4 and Figure 5 indicate that only the rebars embedded in mortar samples of up to 0.4% of Cl^−^ in weight of cement are kept in a passive state. These results are in agreement with the indications of the EHE and ACI [32,33,54]. The embedded rebars in specimens with 0.6% Cl^−^ are maintained with low corrosion levels. Rebars in specimens with 0.8% Cl^−^ are maintained with i_corr_ between 1 and 2 µA/cm^2^. With higher amounts of Cl^−^, the rebars maintain i_corr_ at approximately 10 µA/cm^2^. Similar results were obtained in previous investigations, observing that a greater amount of chloride ion present in the mortar, generates a higher rate of corrosion in the rebars [49,55,56].

All the rebars that have kept the corrosion products (COR) exhibit very high i_corr_ values (10 µA/cm^2^) regardless of the amount of Cl^−^ present in the mortar. Moreover, the i_corr_ of the bars does not depend on the amount of chloride ion, which proves the impossibility of reworking reinforcements with thick corrosion products when these are not eliminated. This is because the i_corr_ in the reinforcements that corrode in an active state are sufficient to maintain an acid pH in the steel/corrosion products interface within such an alkaline medium as concrete, so that, once corrosion has been triggered, Cl^−^ are not necessary to maintain it [49,55,57]. Similar results were obtained by Miranda et al., when they observed that the higher the degree of pre-corrosion of the steel, the higher its corrosion rate, even in chloride-free environments [56,57].

**Figure 6:**
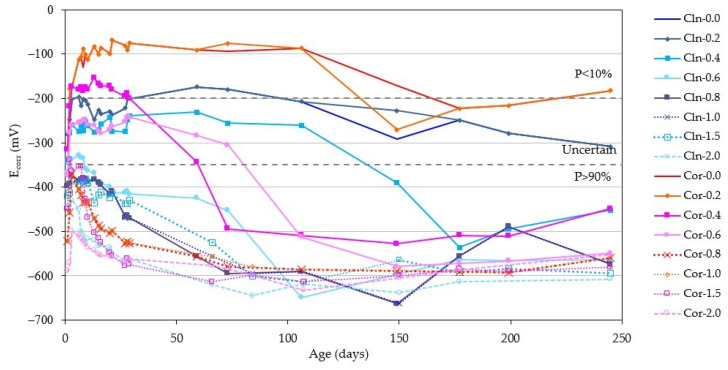
Evolution of the E_corr_ over time for all the rebars studied.

**Figure 7:**
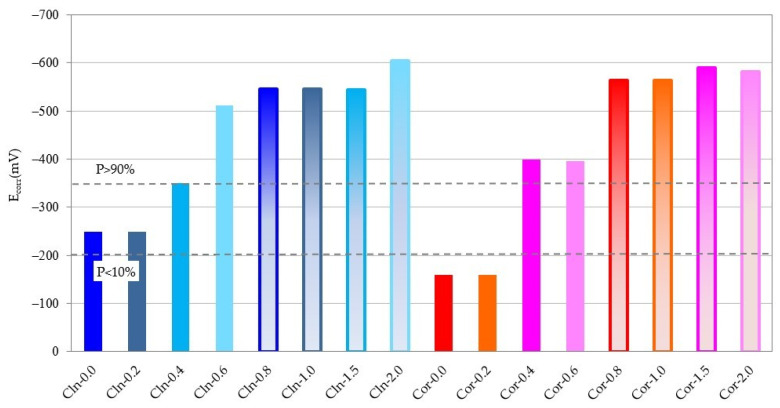
Measurement of the E_corr_ of all rebars after 250 days.

The evolution of the E_corr_ in the same period of time can be seen in Figure 6. Figure 7 depicts in more detail the E_corr_ values reached by all the bars after 250 days.

If we compare the evolution in time of the graphs in Figure 4 and Figure 6, we can see that the E_corr_ suffers a greater variation than the i_corr_, reaching, in some cases (corroded rebar introduced in a specimen with 0.4% of Cl^−^), values, during the whole studied period, typical of the passive, uncertain and active state, since the measurement of the potential for corrosion varies enormously depending on various factors, such as temperature and humidity [58]. It can also be seen that at 250 days, the E_corr_ value depends much more on the amount of Cl^−^ in the specimen than on the passive or active state of the rebar, so that the E_corr_ of rebars in specimens containing the same amount of Cl^−^ are very similar, regardless of whether the rebar has been embedded in the clean specimen or with the corrosion products (Figure 6 and Figure 7).

**Figure 8:**
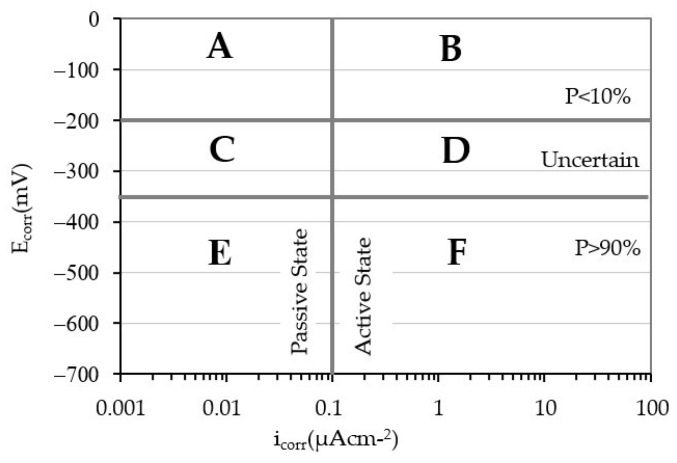
Joint representation of E_corr_ and i_corr_ in the plane.

If the values of E_corr_ and i_corr_ are represented in a system of axes together with the thresholds that delimit the passive and active states, it is possible to check the validity of the E_corr_ measurements as a predictor of the corrosion state of a rebar (Table 3). Choosing as ordinate axis the value of E_corr_ and as *x*-axis the value of i_corr_ and marking by vertical and horizontal lines the thresholds of these values, the space is divided into 6 quadrants (Figure 8).

In quadrants A and F, corrosion states coincide that predict the measurements of the E_corr_ and i_corr_: Quadrant A corresponds to measurements that indicate the passive state while the measurements of quadrant F indicate the active state. In contrast, quadrants B and E define corrosion states with total discrepancy between the prediction of the E_corr_ and i_corr_. Quadrants C and D define corrosion states where the E_corr_ predicts an uncertain state.

**Figure 9:**
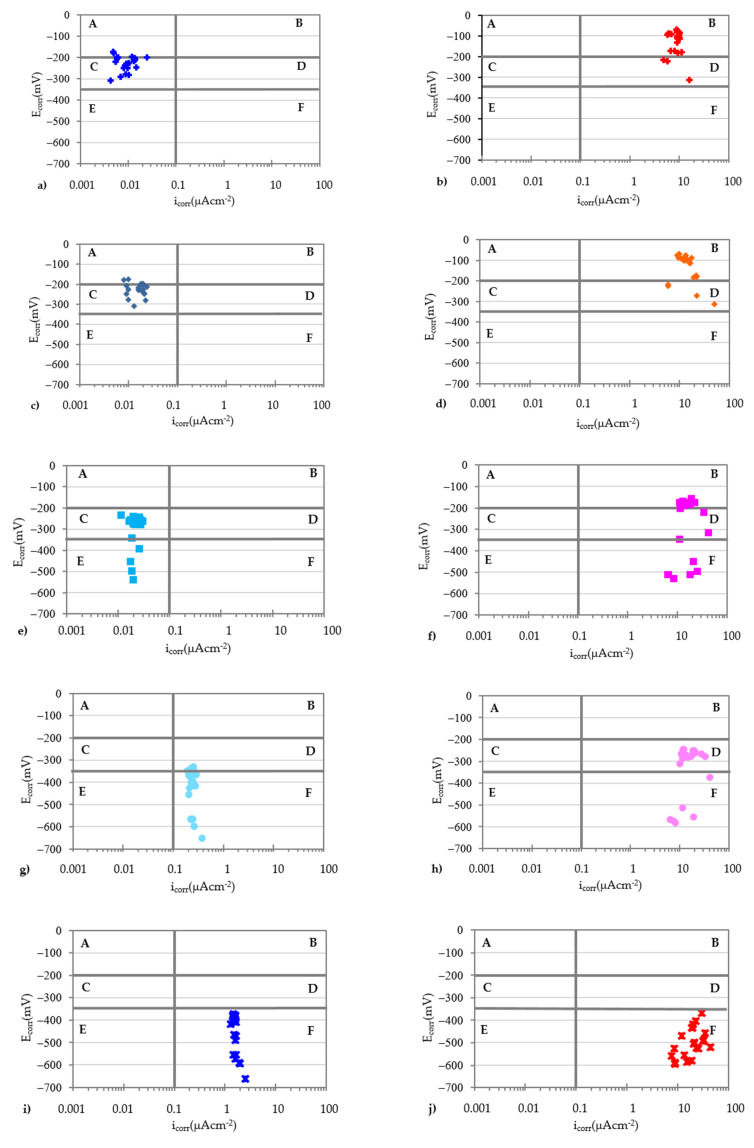

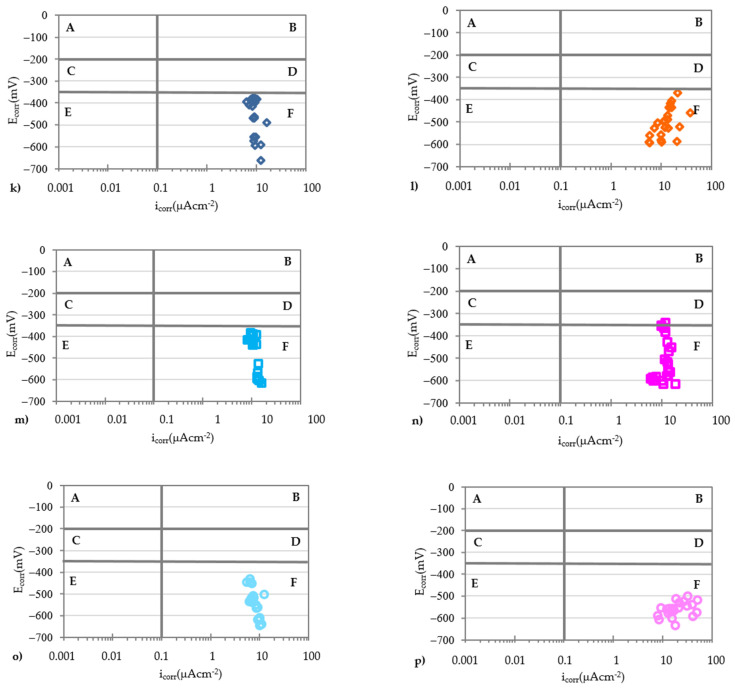
E_corr_ and i_corr_ values for each of the rebars. (**a**) Cln-0.0; (**b**) Cor-0.0; (**c**) Cln-0.2; (**d**) Cor-0.2; (**e**) Cln-0.4; (**f**) Cor-0.4; (**g**) Cln-0.6; (**h**) Cor-0.6; (**i**) Cln-0.8; (**j**) Cor-0.8; (**k**) Cln-1.0; (**l**) Cor-1.0; (**m**) Cln-1.5; (**n**) Cor-1.5; (**o**) Cln-2.0; (**p**) Cor-2.0.

**Figure 10:**
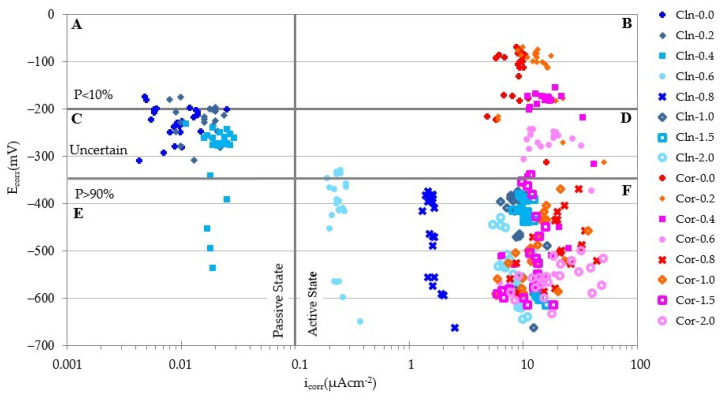
E_corr_ and i_corr_ values for all rebars.

Figure 9 depicts the E_corr_ and i_corr_ values for each of the specimens obtained in all measurements. The graphs on the left column correspond to the clean rebars (CLN) while the right column corresponds to the values of the rebars with corrosion products (COR). In the two graphs located in the same row, the amount of Cl^−^ present in the test pieces coincides. Figure 10 shows the values of all the rebars together.

The analysis of all the graphs in Figure 9, shows that only the predictions of the E_corr_ and i_corr_ for rebars with an ongoing corrosion process and containing at least 0.8% Cl^−^ coincide. In these cases, all the data points of the graphs are located in quadrant F indicated in Figure 8, regardless of whether the rebars are clean or with corrosion products. In addition, the E_corr_ value becomes more electronegative as the amount of Cl^−^ present in the specimen increases and regardless of the state of the rebar. This trend is more pronounced for very low amounts of Cl^−^ and does not depend on the corrosion state of the reinforcement.

The analysis in Figure 10 allows verification of (i) the rebars in passive state (i_corr_ < 0.1 µA/cm^2^) show a large number of E_corr_ values corresponding to high probabilities of corrosion (quadrant A) or uncertain states (quadrant C), and (ii) the rebars in active state (i_corr_ > 0.1 µA/cm^2^) that show a large number of E_corr_ values corresponding to low probabilities of corrosion (quadrant F) or uncertain states (quadrant D). These results show the impossibility of predicting the passive or active state of the steel only by means of E_corr_ measurements [58].

## 4. Conclusions

Previously corroded reinforcing steel bars have been soaked in mortar samples with varying contents of Cl- in two different states: previously pickled and with the corrosion products. They have been kept for 250 days in a humid chamber, and E_corr_ and i_corr_ have been measured and obtained with the following conclusions being drawn:

- All the rebars that have kept the corrosion products showed a very high i_corr_ and a similar value, approximately 10 µA/cm^2^, regardless of the number of chlorides present in the specimen.
- Clean rebars (CLN) embedded in specimens with 0.6 Cl^−^ remain with uncertain i_corr_ (values between 0.1 and 1.0 µA/cm^2^).
- The values of the E_corr_ measurement depended more on the amount of Cl^−^ present in the specimen than on the passive or active state of the rebars.
- Only the predictions of the E_corr_ and i_corr_ coincided in bars embedded in specimens with at least a 0.8% Cl^−^ by weight cement ratio, regardless of whether the rebar is clean or maintains the corrosion products.
- The low correlation of the results obtained in the E_corr_ and i_corr_ in different situations, makes it impossible to predict the passive or active state of the steel solely based on E_corr_ measurements.
- To repair a concrete structure corroded by the effect of chloride ions, the concrete that surrounds the rebars must be removed so that all the corrosion products generated on the surface of the rebars can be eliminated. If the complete removal of the corrosion products is not achieved, even if a repair mortar is placed on it, the rebars will remain in active state.

## Figures and Tables

**Table 1:** Chemical composition of the steel used.

Element	C	Si	Mn	P	S	Cr	Ni	Cu	Mo
Composition (%)	0.21	0.22	0.72	<0.01	0.022	0.05	0.09	0.08	<0.05

**Table 2:** Physical characteristics and chemical composition of cement and sand.

	Physical Characteristics		Chemical Composition	
Cement	Blaine specific surface area	414 m^2^/kg	SO_3_	3.40%
	Density	3.15 g/cm^3^	Cl^−^	0.01%
	Initial setting time	108 min	Calcination loss	1.72%
	Final setting time	160 min	Insoluble residue	0.40%
Sand	Sand equivalent	78	S, SO_3_, Cl^−^ and low specific	
	Real density	2.619 g/cm^3^	weight particles	0.00%
	Normal absorption coefficient	15%	Fine	0.78%
	Saturated surface dry density	2.630 g/cm^3^		
	Clay clumps	0.01%		
	Coefficient of type of course aggregate	0.26%		
	Soft particles	0.93%		

**Table 3:** Reference values for E_corr_ and i_corr_.

Measurement	Risk	Values
E_corr_ (mV)	High >90%	E_corr_ < −350
	Uncertainty	−350 < E_corr_ < −200
	Low <10%	E_corr_ > −200
i_corr_ (μA/cm^2^)	Active state	i_corr_ > 1μA/cm^2^
	High corrosion	0.5 μA/cm^2^ < i_corr_ < 1 μA/cm^2^
	Low corrosion	0.1 μA/cm^2^ < i_corr_ < 0.5 μA/cm^2^
	Passive state	i_corr_ < 0.1 μA/cm^2^

## Data Availability

Data available on request.
